# Interventional Radiology Locoregional Therapies for Intrahepatic Cholangiocarcinoma

**DOI:** 10.3390/life14020217

**Published:** 2024-02-02

**Authors:** Gregory Woodhead, Sean Lee, Lucas Struycken, Daniel Goldberg, Jack Hannallah, Shamar Young

**Affiliations:** 1 Department of Medical Imaging, Division of Interventional Radiology, University of Arizona Medical Center, Tucson, AZ 85712, USA; lstruycken@radiology.arizona.edu (L.S.); dgoldberg@radiology.arizona.edu (D.G.); jackh@arizona.edu (J.H.); shamar@arizona.edu (S.Y.); 2 Department of Basic Biomedical Sciences, Touro College of Osteopathic Medicine, Middletown, NY 10027, USA; slee55@student.touro.edu

**Keywords:** intrahepatic cholangiocarcinoma, Y90, SIRT, TACE, cryoablation, microwave ablation, radiofrequency ablation, irreversible electroporation, interventional radiology, locoregional treatment

## Abstract

Surgical resection remains the cornerstone of curative treatment for intrahepatic cholangiocarcinoma (iCCA), but this option is only available to a small percentage of patients. For patients with unresectable iCCA, systemic therapy with gemcitabine and platinum-based agents represents the mainstay of treatment; however, the armamentarium has grown to include targeted molecular therapies (e.g., FGFR2 inhibitors), use of adjuvant therapy, liver transplantation in select cases, immunotherapy, and locoregional liver-directed therapies. Despite advances, iCCA remains a challenge due to the advanced stage of many patients at diagnosis. Furthermore, given the improving options for systemic therapy and the fact that the majority of iCCA patients succumb to disease progression in the liver, the role of locoregional therapies has increased. This review will focus on the expanding role of interventional radiology and liver-directed therapies in the treatment of iCCA.

## 1. Introduction

Cholangiocarcinoma (CCA) is the second most common primary liver malignancy, and its incidence, currently at 2.1 per 100,000, is rising [1,2]. CCA is not one class of malignancy; instead, it represents a group of epithelial tumors arising from the biliary tree that confer a poor prognosis, notable for a five-year mortality rate of 95% [3,4,5,6]. Classification is based on anatomic location within the biliary tree and biological characteristics, including intrahepatic cholangiocarcinoma (iCCA), perihilar cholangiocarcinoma (pCCA), distal cholangiocarcinoma (dCCA), and cholangiocarcinoma arising from the gallbladder proper. pCCA typically arises in the perihilar biliary system, representing the so-called Klatskin tumor. dCCA involves the distal/extrahepatic common bile duct. By comparison, iCCA arises from the intrahepatic biliary system, typically as a mass within the hepatic parenchyma [5]. Compared to pCCA and dCCA, iCCA is frequently detected incidentally, as it is most commonly asymptomatic. While surgical resection can be curative for iCCAs that are caught early, greater-than-90% of patients are deemed unresectable at diagnosis [7,8]. Furthermore, the recurrence rate after attempted curative resection is high with a meta-analysis, indicating an incidence of approximately 70% [9,10]. Due to these factors, among others, of the three subtypes of CCA, iCCA has the worst prognosis. To further complicate the matter, some minority of lesions show a mixed or biphenotypic appearance. That is, they represent some combination of characteristics of both cholangiocarcinoma and hepatocellular carcinoma (HCC). Very little is known about the optimal treatment strategies for this subclass of tumors [11], and therefore the focus of this review will be on iCCA.

Systemic therapies have traditionally been the mainstay of treatment for unresectable CCA, but unfortunately, the prognosis remains poor. Evaluation of new therapies for cholangiocarcinoma has been somewhat limited, secondary to challenges with developing strong small animal models of iCCA and the relative rarity of the disease [12]. However, there have been advances in systemic therapy which have coincided with the rise of immunotherapies. This is most clearly seen in the success of the TOPAZ-1 trial, which supports the addition of Durvalumab to gemcitabine and cisplatin as first line therapy [13]. However, multiple other targeted therapies are being evaluated in this patient populations [14]. Despite these advances the outcomes are still less than desirable, and the majority of patients ultimately die from local advancement in the liver. This has increased the interest in adding locoregional therapies to the treatment algorithms [15]. Despite initial concern that the combination of systemic therapies and locoregional therapies may confer a high toxicity rate, early data have supported its safety [16].

In this review, we explore the evidence for locoregional therapies with a strong emphasis on locoregional therapies performed by interventional radiology in patients with iCCA.

### 1.1. Locoregional Therapies within Context and Staging of iCCA

While staging of iCCA has been thoroughly discussed elsewhere, the authors provide a brief review to set the stage for the coming discussion. The updated 8th edition AJCC/UICC guidelines are briefly reviewed with the staging as follows [17]: Stage I iCCA is further subclassified as Stage IA, a single tumor less than 5 cm, or Stage IB, a single tumor greater than 5 cm [17]. Stage II iCCA disease is characterized by vascular invasion, or the presence of more than one tumor [17]. Stage III iCCA is again further subdivided into Stage IIIA, defined by a tumor spread through the liver capsule, or Stage IIIB, local extra-hepatic spread (i.e., periportal lymph nodes) [17]. Finally, Stage IV iCCA is characterized by distant metastatic disease (i.e., lungs) [17].

The heterogeneity of the tumors evaluated in various studies cannot be overstated [18]. For instance, evaluations into locoregional therapy have included patients in the first line stage IA setting, patients with recurrence after prior curative intent therapy, and disease control in advanced stage disease are just a few examples of the many scenarios highlighted below. This heterogeneity has made interpretation of the efficacy of IR liver-directed treatments challenging and is further complicated by the fact that often these varying scenarios are presented in a single paper.

### 1.2. Non-IR Local-Region Treatment Options for iCCA

As an aside, there are several types of locoregional therapy for iCCA that are not performed by IR. The role of external beam therapy (EBT), including proton beam therapy (PBT) and stereotactic body radiation therapy (SBRT), and the hepatic arterial infusion (HAI) of chemotherapy have been discussed elsewhere. While data for the use of HAI are lacking, data for the value of EBT as a locoregional approach to iCCA are promising in well-selected patients [19,20,21,22,23,24,25,26].

### 1.3. IR Locoregional Therapies for iCCA

#### 1.3.1. Thermal Ablation: RFA, MWA, Cryoablation

Thermal ablation is a general category of locoregional therapies that includes both heat (radiofrequency ablation (RFA) and microwave ablation (MWA)), as well as cold (cryoablation)-based techniques. Although newer ablation technology often does not rely on thermal destruction of cells, such as irreversible electroporation (IRE), they are often grouped into this category as well. These various techniques share in common that they typically involve the placement of one or more needles within a target tumor which delivers energy intended to destroy the tumor cells adjacent to the needle. Given the difference in underlying cell death mechanisms, data from one form of ablation are not necessarily applicable to other forms, and so each is discussed separately here.

#### 1.3.2. Radiofrequency Ablation (RFA) of iCCA

RFA is the most studied thermal based ablation technique and utilizes high-frequency alternating electric current to induce cell death via rapid electron vibration, in turn generating frictional heat. Accordingly, RFA depends on conductivity of the target parenchyma, which largely correlates with water content of that tissue. Therefore, a limitation of RFA is that tissue desiccation induces an insulating sleeve and thus hinders further propagation of the heat energy, limiting ablation zone size. RFA is also prone to heat sink, which is the cooling effect induced by nearby flowing blood in hepatic or portal veins. In order to generate large ablation zones, multiple probes are utilized. Along with MWA (discussed below), RFA is a heat-based thermal ablation technique. Typically, because of these limitations, RFA is considered only in patients with relatively small tumors.

As the most established form of percutaneous ablation, many studies have evaluated the use of RFA for iCCA. While no randomized trial has been published, multiple retrospective studies [27,28,29,30,31,32,33,34,35,36,37] and one prospective cohort study have evaluated the use of RFA for patients with iCCA [38]. Unfortunately, the existing studies generally suffer from small tumor/patient numbers and are heterogeneous in design and patient/disease factors.

Below, we will review a few of the most impactful studies in this area. The first study to demonstrate successful use of RFA to treat iCCA was published in 2005 [38]. It is important to note that RFA technology evolved over the following decade such that the subsequent seven publications are likely more reflective of modern RFA devices. Overall, these studies are retrospective in design and involve a very small number of patients, ranging from 6–29 patients [27,28,29,31,32,33]. These small numbers are likely attributable to the advanced stage at which most iCCA is diagnosed in most patients, excluding percutaneous ablation as a suitable approach for management. However, they do suggest the safety of the approach with the main reported complications (ascites or pleural effusions, liver abscesses, portal vein thrombosis, jaundice, and hepatic failure) occurring infrequently and the rate of severe complications being low overall.

The largest study of RFA for iCCA evaluated 20 patients with a total of 50 tumors [35], of which, 44 tumors were treated with RFA. The median OS was 23.6 months and RFA was found to be safe and effective, with no major complications reported. The most recent study involved 20 patients who had iCCA in the setting of cirrhosis [34]. As in the prior publication by Takahashi et al., RFA for iCCA was found to be effective and safe in this patient population, reflecting similar data from the HCC population. A recent systemic review of locoregional therapy suggested that RFA achieves adequate local control of iCCA. Further, the “response rate” was calculated to be 93.9% [19].

While limited by low power and heterogeneity, these studies suggest two factors that correlate with improved outcomes following RFA of iCCA: (1) increasing treatment margins, and (2) smaller tumors selected for treatment. For example, in a small study of seven patients with nine iCCAs ranging in size from 1.3 cm to 33.3 cm (mean: 2.4 cm), which demonstrated a promising mean OS of 38.5 months [27], the authors reported intentionally inducing a larger ablation zone, relative to typical ablation margins when treating comparable-sized HCCs in their practice. They speculated that a larger ablation margin may overcome the more infiltrative nature of iCCAs. A subsequent meta-analysis of seven RFA studies of unresectable primary and recurrent iCCA, which reported a median OS of 20 to 60 months and a combined 1-, 3-, and 5-year survival of 82, 47, 24%, respectively [39], recognized that the size of the tumor was in part responsible for treatment success. Similarly, Bradi et al. suggested that a tumor size less than 2 cm is an independent factor for improved local tumor progression-free survival [33]. Seeming to support this assertion, Diaz-Gonzalez et al. found that the median time to recurrence was significantly lower when the tumor was less than 2 cm [34].

#### 1.3.3. Microwave Ablation (MWA) of iCCA

Although a newer form of heat-based thermal ablation, MWA has quickly supplanted RFA in the majority of liver-directed treatments, in many geographic areas, due to its ability to generate a large ablation volume with a smaller number of probes and its decreased tendency to suffer from heat-sink [40]. Microwave technology deposits energy into tissues through electromagnetic radiation-induced rotation of dipole molecules, namely water, resulting in frictional heating [41]. As such, MWA generates higher temperatures than RFA, contributing to larger ablation zones and is more effective in tissues with greater impedance.

While a smaller number of total studies has been published using MWA for iCCA, these studies tend to include more patients than those discussed above evaluating RFA [34,35,42,43,44,45]. Unfortunately, as with RFA, there are no randomized prospective studies that investigate outcomes after MWA of iCCA; accordingly, the retrospective design of MWA studies leads to difficulties with cross-trial comparison. Also, as with the RFA data, the available MWA studies suffer from the same heterogeneity of patients/tumors and clinic scenarios.

The largest such study utilizing MWA in iCCA patients retrospectively evaluated 107 patients with 177 Stage I tumors (maximum of 3 tumors per patient), constituted by a combination of primary and recurrent iCCA patients, who underwent MWA [46]. This study achieved an OS at 1, 3, and 5 years of 93.5, 39.6, and 7.9%, respectively. Seeming to show parallel findings with RFA [28], patients with fewer and smaller tumors at the start of treatment showed better outcomes [46].

Two studies have tried to compare MWA to surgical resection in patients with small iCCAs. First, Zhang SJ et al. evaluated outcomes for 109 patients with recurrent Stage IA iCCA who underwent either RFA and/or MWA vs. repeat resection [45]. The median OS at 1, 2, and 3 years was not significantly different between the ablation (69.8, 37.3, and 20.5%) and resection (83.8, 38.0, and 17.1%) cohorts [45]. Subsequently, MWA was compared with surgical resection in 121 patient’s with primary Stage IA iCCA (56 MWA, 62 surgical resection; median tumor size 2.6 cm) [44]. Again, no significant difference was found between the OS at 5 years, 23.7% vs. 21.8%, respectively, and it is notable that the MWA patient group had an overall lower performance status (mean Karnofsky 35 vs. 60) suggesting there was a bias to select sicker patients for MWA.

#### 1.3.4. Cryoablation of iCCA

**Figure 1:**
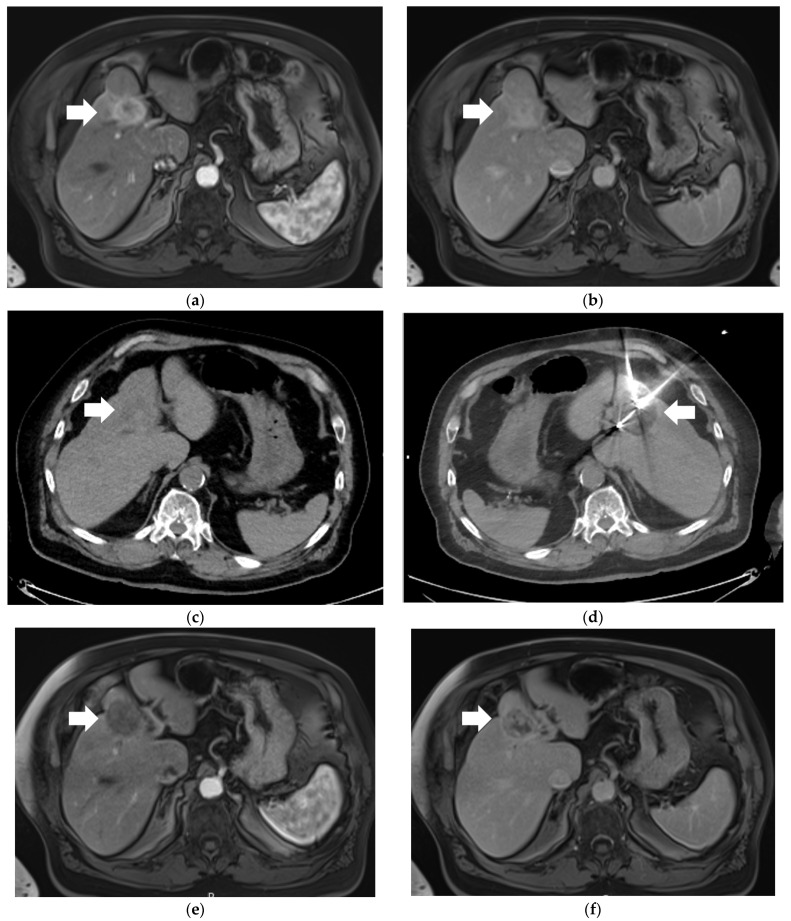
77-year-old male with a 3.7 cm intrahepatic cholangiocarcinoma (iCCA) in Segment 4A of the liver who underwent percutaneous cryoablation. (**a**) Selected arterial-phase contrast-enhanced T1-weighted magnetic resonance imaging (MRI) image demonstrating avid enhancement of the iCCA (arrow) compared to background liver parenchyma. (**b**) Selected delayed-phase contrast-enhanced T1-weighted MRI image demonstrating persistently elevated enhancement of the iCCA (arrow) relative to background liver. (**c**) Selected pre-procedural non-contrast axial CT image demonstrating the iCCA (arrow) as hypodense relative to background liver parenchyma. (**d**) Selected intra-procedural non-contrast axial CT image demonstrating two of a total of three cryoablation probes positioned within the iCCA, as well as associated “ice ball” (arrow). (**e**) Selected 1-month post-cryoablation arterial-phase contrast-enhanced T1-weighted MRI image demonstrating absence of previously seen tumoral enhancement. (**f**) Selected 1-month post-cryoablation delayed-phase contrast-enhanced T1-weighted MRI image demonstrating absence of previously seen tumoral enhancement.

Cryoablation leads to cell death due to cell membrane and organelle damage caused by dehydration and osmotic pressure changes from the formation of intra- and extracellular ice crystals [47,48]. High pressure argon gas (3000 psi) is allowed to travel along the length of the ablation needle to the tip, where it subsequently expands rapidly to atmospheric pressure. This rapid expansion results in profound cooling at the tip of the ablation needle via the Joule–Thomson effect, reaching temperatures as cold as −160 °C. One technical advantage of cryoablation is the ability to visualize the ice ball formation in real-time either by MRI, CT, or US, which aids in determining tumor coverage and treatment margins. It is this ability to visualize the ice ball that makes cryoablation a suitable alternative to hyperthermal techniques when targeting liver tumors that are close to delicate structures, such as the gallbladder or loops of bowel. An example of a Segment 4B iCCA that was in close proximity to the hepatic flexure colon which was treated by percutaneous cryoablation is provided (Figure 1). Patients undergoing cryoablation report less pain both intra- and post-procedure, relative to MWA or RFA [49]. This may in part reflect analgesic properties of cryoablation but is a reminder of the nociceptive pain experienced during and after heat-based thermal ablation procedures. Accordingly, cryoablation may provide an operational/workflow advantage, as these procedures are routinely performed with moderate sedation only. Possible disadvantages of cryoablation include the fact that it can be more time consuming and multiple probes may be necessary to generate a treatment zone of a desired size, as well as the increased cost that using multiple probes entails. A rare but major complication is cryoshock, which has been reported to occur in 0.3–2.0% of cases, particularly during treatment of liver tumors [50,51].

There are no current studies designed to specifically evaluate the effectiveness of cryoablation for iCCA. One single center reviewed cryoablation of 299 primary and metastatic liver tumors, but only 6 were iCCA [52]. Another study of cryoablation of liver tumors included a total of thirty-nine patients, but only three were iCCA [53]. These early studies proposed that cryoablation of iCCA is safe and effective; however, no long-term OS data was collected or evaluated.

There is significant need to develop either retrospective or prospective studies to evaluate the safety and feasibility of cryoablation for iCCA, and learn about any unique advantages, limitations, and/or risks. From extension from observations made by abovementioned studies of RFA for Stage 1 iCCA, we might anticipate using more cryoprobes to generate oversized ice balls to achieve larger treatment margins will contribute to greater tumor control. Ultimately, cryoablation may play a limited by essential role in the treatment of iCCAs in specific locations (e.g., adjacent to delicate structures, including nerves or innervated structures), specific subsets of patients who cannot tolerate RFA/MWA or surgical resection (e.g., poor general anesthesia candidates, as most patients can tolerate cryoablation under conscious sedation), or for specific sizes (e.g., very small proximal tumors that result in peripheral segmental biliary obstruction). Therefore, based on extrapolation from HCC or treatment of mCRC, we might expect that cryoablation will find a role for treating iCCAs in locations that are higher risk of injuring delicate adjacent structures, e.g., neighboring loops of bowel. As a final note, extrapolation of data from small animal and human studies that have looked at cryoablation and immunomodulation: cryoablation is broadly believed to induce significantly more adaptive immune education/priming vs. heat-based thermal devices [54,55,56]. The difference in immunomodulation may be in part due to the fact that cryoablation preserves the tumoral antigens, which can then serve as priming agents for the immune system [57].

### 1.4. Non-Thermal Ablation: IRE, Histotripsy

#### 1.4.1. IRE of iCCA

Irreversible electroporation is a non-thermal based ablation technology that incudes permeabilization of cell membranes via application of a high-voltage, low energy direct current (DC), also known as a pulsed electric field (PEF) [58]. Delivery of high voltage electrical current (up to 3000 V) between probes creates nanoscale holes (80–490 nanometers) in cell membranes [58]. Cells within the ablation zone lose the ability to maintain homeostasis and therefore undergo apoptotic cell death in a narrow zone of transition [59]. Due to their unstable membrane structure, IRE drives tumor-specific membrane damage/cell death while sparing the adjacent stromal cells [60]. As it is non-thermal, IRE does not suffer from heat or cold sink, which are limitations of RFA/MWA and cryoablation, respectively [59]. Additionally, IRE can be considered for tumor adjacent to sensitive structures (gallbladder, major bile ducts, and bowel loops) [61,62].

One limitation of IRE is that the high voltage delivered causes muscular contraction, raising the potential for cardiac arrythmia [63]. Therefore, IRE must be performed under general anesthesia with complete neuromuscular blockade and electrocardiogram synchronization [64]. Traditionally, several practical limitations prevented the broad adoption IRE, including the need for multiple probes, requirements of probes being precisely aligned, and the high cost of each probe [60]. However, new developments in IRE are underway that may address these challenges.

Overall, there is a lack of data evaluating the use of IRE for iCCA, due in part to the novelty of IRE and rarity of unresectable primary or recurrent iCCA suitable for treatment with IRE. A 2017 systematic review and meta-analysis of IRE for hepatic tumors included nine studies with three-hundred patients, but only twenty-one had iCCA and no subgroup analysis for iCCA was performed [65]. Recently, a prospective feasibility study evaluated IRE for a total of fifteen patients with unresectable CCA, representing eight iCCA and seven pCCA tumors [66]. No major complications were observed. For the iCCA group, follow up ranged from 6–36 months and 50% survived at time of last follow. A Kaplan–Meier analysis showed a mean survival of 18 months with size of the original iCCA tumor appearing to predict response [66]. In a more recent prospective pilot study, Franken et al. evaluated IRE for treatment of pCCA. They reported promising results: median OS from diagnosis was 21 months with a 1-year survival rate of 75% after IRE [67]. While we cannot directly extrapolate from this study to iCCA, it may serve as a signal, and it reinforces the need to develop prospective (likely multicenter) studies of IRE for iCCA.

#### 1.4.2. Histotripsy of iCCA

An emerging non-thermal approach to target tumor tissue disruption is histotripsy (Histosonics). Histotripsy is a noninvasive, nonionizing, and nonthermal focused ultrasound ablation method that is currently being developed for the treatment of liver cancer [68]. No trial is currently underway for histotripsy for iCCA; however, the company has launched preliminary small animal and ex vivo tumor studies. Of note, histotripsy was successfully used to ablate iCCA tumors in vivo using a patient-derived xenograft mouse model [69]. As noted above for cryoablation, non-thermal tissue disruptive forms of ablation (IRE and histotripsy) may prove to be better in combination therapy with ICIs, as tumor-specific antigens should be preserved.

### 1.5. Transarterial Chemoembolization (TACE) of iCCA

TACE has been commonly utilized in iCCA patients, largely because of the familiarity with using TACE for HCC. When performing a TACE procedure, a microcatheter is utilized to select the specific hepatic artery or arteries supplying an iCCA. Once selected, a mixture of embolic agent and chemotherapy is administered. TACE comes in two forms: conventional TACE (cTACE) and drug-eluting bead TACE (DEB-TACE). In cTACE, lipiodol serves as the chemotherapy carrying agent and this is followed by an embolic. In DEB-TACE the chemotherapy is loaded onto the bead/sphere which also works as the embolic agent. High concentration chemotherapy is mixed directly with lipiodol for cTACE. In contrast, precisely sized beads are preloaded with chemotherapy by the pharmacy prior to delivery in DEB-TACE. In both forms, the idea is to deliver a high dose of chemotherapy directly to the tumor while also inducing ischemia.

Unfortunately, as with the ablation literature, most studies reported to date are limited by retrospective design and small patient numbers. These studies also suffer from lack of standardization regarding chemotherapy drugs used and conventional versus DEB-TACE technique, resulting in heterogeneous data. One retrospective study that is notable for its relatively large patient number (n = 127) showed that DEB-TACE was safe for iCAA with a good disease control rate of 95%, which constituted partial response (15%) or stable disease (80%) [70].

To date, only one randomized clinical trial (n = 48) has evaluated TACE plus systemic chemotherapy versus chemotherapy alone in patients with advanced iCCA [71]. This trial demonstrated improvement in progression free survival of 20 months in the combination arm [71]. Further, the median survival was 12.2 months in the TACE group and 3.3 months in the control group. Unfortunately, more than 50% of patients in both groups went on to develop extra-hepatic metastasis. A separate Phase 2 trial (n = 50) reported an improved median OS for patients in favor of gemcitabine/cisplatin plus DEB-TACE versus DEB-TACE alone (median OS 33.7 [95% CI 13.5 to 54.5 months] versus 12.6 months [95% CI 8.7 to 33.4 months (*p* = 0.048)] [72]. Patients with CP greater or equal to 1, hypervascular tumors, large tumor size (greater than 5 cm), or multifocal tumors demonstrated worse outcomes [72]. Thus, this Phase 2 study may support a role for TACE in carefully selected iCCA patients.

Overall, TACE procedures are generally well tolerated. Grade 3–4 AEs are seen in around 10% of patients but can reach 25% of patients in some studies [70,71,73,74,75,76,77,78,79]. Therefore, for multifocal iCCA with a tumor burden of less than 50% of liver parenchyma patients with good performance status (PS) less than two, and good liver function CP A5-6 or B7, TACE may achieve local control of tumor growth.

### 1.6. Selective Internal Radiation Therapy (SIRT) and iCCA:

SIRT is a radiation brachytherapy which delivers small (20–60 µm) particles that carry the isotope yttrium-90 (Y90). Y90 is a beta-emitter with an average energy of 0.94 megaelectron volts (half-life of 2.67 days), decaying to zirconium-90. Y90 penetrates soft tissues to an average depth of 2.5 mm (maximum 11 mm) [80,81]. Y90 particles are delivered to hepatic tumors via transarterial approach [81]. The treatment consists of two procedures. The first procedure is called the mapping and consists of using catheters to identify the arteries that supply the tumor. A test particle, technetium 99 m macroaggregated albumin (MAA), is then delivered to determine distribution within the treatment area and measure lung shunt fraction [80]. The patient then returns to have the Y90 microspheres delivered. This procedure has gained wide acceptance in treatment of primary and secondary cancers of the liver over the last several decades [82]. Unfortunately, the majority of SIRT studies available suffer from the same limitations as discussed previously.

As with TACE, Y90 SIRT for iCCA has been variably evaluated. Multiple single-center and multicenter retrospective studies have evaluated Y90 SIRT for iCCA [83,84,85,86,87,88,89,90,91,92,93]. Y90 SIRT has been performed alone or in conjunction with systemic chemotherapy [16,94,95]. A notable recent multicenter retrospective study evaluated Y90 SIRT for patients (n = 81) with unresectable iCCA in three groups: (A) chemotherapy naïve, (B) disease control after first-line chemotherapy, and (C) progression after first-line chemotherapy [96]. This study found that the median OS was not significantly different across these groups at 14.5 months (95%: 11.1–16.9). Instead, the median OS appeared to be determined by tumor size/extension, N/L ratio, and radiological response rates after Y90 [96].

Only one prospective clinical trial (n = 41) to date has evaluated Y90 SIRT with concomitant chemotherapy [16,97]. Results from the first-line single-arm MISPHEC study suggest that gemcitabine/cisplatin with SIRT for patients with unresectable iCCA can achieve a high disease control rate of 98% (95% CI, 80–99%) at 3 months, with median PFS of 14 (95% CI, 8–17) months, and median OS of 22 (95% CI, 14–52) months [16]. Notably, 9/41 (22%) of patients were down staged to curative intent surgery.

Recently, Edeline et al. performed a retrospective analysis of data from patients enrolled in MISPHEC with the corresponding data from patients enrolled in previous prospective trials who were treated with first-line chemotherapy alone (ABC-01, ABC-02, ABC-03, BINGO, and PRODIGE 38 AMEBICA) [97]. Utilizing an emulated target trial paradigm and inverse probability of treatment weighting methods, this analysis suggests that Y90 SIRT combined with chemotherapy might improve outcomes over chemotherapy alone in patient with liver-only iCCA.

Furthermore, Y90 SIRT has been compared with TACE for the treatment of unresectable iCCA in a single institution retrospective analysis, which demonstrated similar toxicity and disease control in this population [73].

Finally, a recent single-center retrospective study has evaluated the role for Y90 SIRT plus systemic chemotherapy to downstage locally advanced iCCA in order to permit curative intent surgical resection [98]. In this study, thirteen patients with unresectable iCCA underwent three cycles of adjuvant gemcitabine/cisplatin, followed by Y90 SIRT plus capecitabine, and then another three cycles of gemcitabine/cisplatin, prior to restaging. Ultimately, 7/13 patients were successfully downstaged to curative-intent resection. While small sample size limits a thorough analysis of this institutional approach, this represents a very attractive treatment algorithm as the concept of downstaging to curative intent therapy has not previously been realistic in this disease process.

Given the increasing role of immunotherapy in iCCA and the fact that radiation is known to help prime the immune system, there is significant interest in evaluating combined treatment techniques [99,100,101]. This underlies the importance of establishing the safety of the combined systemic therapy and SIRT treatment strategy.

## 2. Future Strategies

### Combination Therapies: Locoregional Therapy in Conjunction with Immunotherapy

As referenced above, the future of iCCA treatment will most likely hinge on combination therapies, i.e., liver-directed locoregional in conjunction with systemic and specifically immunotherapy. A theoretical limitation of any heat-based thermal ablative approach may be the denaturing of tumor-specific antigens, such that any possible immune education/priming may be reduced vis à vis other treatment strategies. Additionally, RFA is believed to induce upregulation at the peripheral of the ablation zone, where non-lethal heating occurs, possibly via the upregulation of HIF1alpha [102]. Accordingly, focusing on developing data around the ability of non-heat based thermal ablation (cold or non-thermal), TACE, and SIRT is likely of great importance and interest. Of these, cryoablation, IRE, and SIRT have perhaps garnered the most interest. This in part is secondary to preclinical studies which vouch for the ability of these locoregional therapies to positively modulate the immune system. Therefore, more data in these arenas are sorely needed.

## 3. Conclusions

The role of IR locoregional therapies in the treatment of iCCA is expanding and early data seems to signal this will confer improved treatment outcomes for patients. At this time, liver-directed therapy provided by IRs may be of value for the treatment of iCCA in several clinical scenarios (Table 1). Notably, ablation or super selective Y90 or TACE may be helpful for patients with Stage IA iCCA who are not candidates for surgical resection. Additionally, the early data are promising for Y90 SIRT in combination with neoadjuvant chemotherapy for the downstaging of locally advanced iCCA prior to surgical resection. However, more data, particularly in the form of prospective studies and in the realm of combination treatment strategies, are sorely needed.

## Figures and Tables

**Table 1:** Clinical scenarios in which interventional radiology locoregional treatments may be beneficial for patients with intrahepatic cholangiocarcinoma (iCCA). While surgical resection offers patients with iCCA the best opportunity for cure, there are many cases in which resection may not be an option. (a) In cases in which an iCCA arises in a location that precludes surgery or in a patient who is not a safe operative candidate (e.g., cirrhotic with portal hypertension), then percutaneous ablation or super selective Y90 or TACE may be considered with a goal of achieving local control or even curative intent for smaller tumors—i.e., Stage 1A (single tumor < 5 cm). (b) Locally advanced iCCA with bilobar spread may be considered for Y90 or TACE in order to achieve control in the liver. Furthermore, based on institutional protocols, some patients with locally advanced iCCA with unilobar spread may be candidates for neoadjuvant Y90 or TACE combined with chemotherapy in order to downstage patients to surgical resection. (c) In addition to receiving systemic chemotherapy, patients with metastatic iCCA may be considered for interventional radiology locoregional treatments in the appropriate setting with a goal of achieving control in the liver.

	(a)	(b)	(c)
	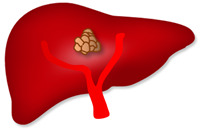	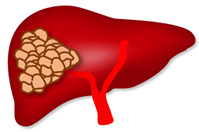	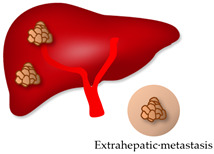
Clinical Scenario	Unresectable iCCA or nonsurgical patient	Locally advanced iCCA	Metastatic iCCA
IR Locoregional Treatment	Percutaneous ablation (RFA/MWA, cryo, or IRE) vs super-selective Y90 or TACE	Y90 or TACE	May be appropriate depending on the size/number of iCCA
Goal(s)	Local control or curative intent	Local control or downstaging as bridge to surgical resection	Achieve control in the liver
